# Mitophagy impairment is involved in sevoflurane-induced cognitive dysfunction in aged rats

**DOI:** 10.18632/aging.103673

**Published:** 2020-09-09

**Authors:** Yeru Chen, Piao Zhang, Xianyi Lin, Huan Zhang, Jiamin Miao, Youfa Zhou, Gang Chen

**Affiliations:** 1 Department of Anesthesiology, Sir Run Run Shaw Hospital, School of Medicine, Zhejiang University, Hangzhou 310058, Zhejiang, China

**Keywords:** sevoflurane, postoperative cognitive dysfunction, mitophagy, aged rat, rapamycin

## Abstract

Postoperative cognitive dysfunction (POCD) is frequently observed in elderly patients following anesthesia, but its pathophysiological mechanisms have not been fully elucidated. Sevoflurane was reported to repress autophagy in aged rat neurons; however, the role of mitophagy, which is crucial for the control of mitochondrial quality and neuronal health, in sevoflurane-induced POCD in aged rats remains undetermined. Therefore, this study investigated whether mitophagy impairment is involved in sevoflurane-induced cognitive dysfunction. We found sevoflurane treatment inhibited mitochondrial respiration and mitophagic flux, changes in mitochondria morphology, impaired lysosomal acidification, and increased Tomm20 and deceased LAMP1 accumulation were observed in H4 cell and aged rat models. Rapamycin counteracted ROS induced by sevoflurane, restored mitophagy and improved mitochondrial function. Furthermore, rapamycin ameliorated the cognitive deficits observed in aged rats given sevoflurane anesthesia as determined by the Morris water maze test; this improvement was associated with an increased number of dendritic spines and pyramidal neurons. Overexpression of PARK2, but not mutant PARK2 lacking enzyme activity, in H4 cells decreased ROS and Tomm20 accumulation and reversed mitophagy dysfunction after sevoflurane treatment. These findings suggest that mitophagy dysfunction could be a mechanism underlying sevoflurane-induced POCD and that activating mitophagy may provide a new strategy to rescue cognitive deficits.

## INTRODUCTION

Postoperative cognitive dysfunction (POCD) is a severe central nervous system complication characterized by impaired memory, a decreased ability to process information and anxiety [1, 2]. Advanced age is one of the major risk factors for POCD, and elderly people experiencing cognitive dysfunction lose the capacity to accomplish daily tasks and live independently [3, 4]. Therefore, it is of interest to understand the mechanisms underlying POCD. Accumulating evidence indicates that inhalation anesthesia elicits neurotoxicity and neurocognitive decline in elderly patients experiencing POCD [5, 6]. Our previous work with an aged rat model of POCD suggested that inhalation anesthesia with sevoflurane causes learning and memory deficits by inducing neuronal apoptosis. [7]. Some studies have demonstrated that immune inflammation contributes to neurotoxicity and cognitive dysfunction in aged rats [8, 9]. Nevertheless, the pathological mechanisms of POCD have not been fully elucidated, and the current methods for preventing and treating POCD are quite limited.

Mitochondria play a critical role neuronal homeostasis by supplying energy and deciding cell survival. Poor mitochondrial quality has been intimately linked with a variety of neurodegenerative disorders [10]. Likewise, mitochondrial dysfunction is increasingly considered a significant contributor to POCD [11, 12]. Induction of POCD in elderly rats is accompanied by an increase in oxidative stress, mitochondrial dysfunction and reduced neurotrophic levels [13]. In addition, increased levels of mitochondria-derived reactive oxygen species (ROS) are closely associated with neuroinflammation in POCD [9]. These studies indicated that mitochondria, which are indispensable for neuronal survival and mitochondria quality control, play an important role in POCD progression. However, it remains undetermined how mitochondrial quality in neurons is controlled during POCD.

Dysfunction in autophagic flux, which mainly includes the formation of autophagosomes and clearance of cargo, has also been linked to neurodegenerative diseases [14–16]. It is well accepted that autophagy is the principle machinery for eliminating damaged or superfluous mitochondria, which is termed mitophagy [17]. Mitophagy reduces cellular stress triggered by increased oxidative stress and thus plays a crucial role in neurodegenerative disease and aging [18–20]. At the molecular level, the PINK1/Parkin pathway has been implicated in mitophagy induction [21, 22]. Our previous study demonstrated that autophagy dysfunction is involved in sevoflurane-induced cognitive deficit of aged rats [23]. However, it remains unclear whether impaired mitophagy is involved in the pathogenesis of POCD in aged rats’ model. In the present study, we tested the hypothesis that neuronal mitophagy plays an important role in the cognitive deficits observed after sevoflurane administration.

## RESULTS

### Sevoflurane induced mitochondria impairment

**Figure 1:**
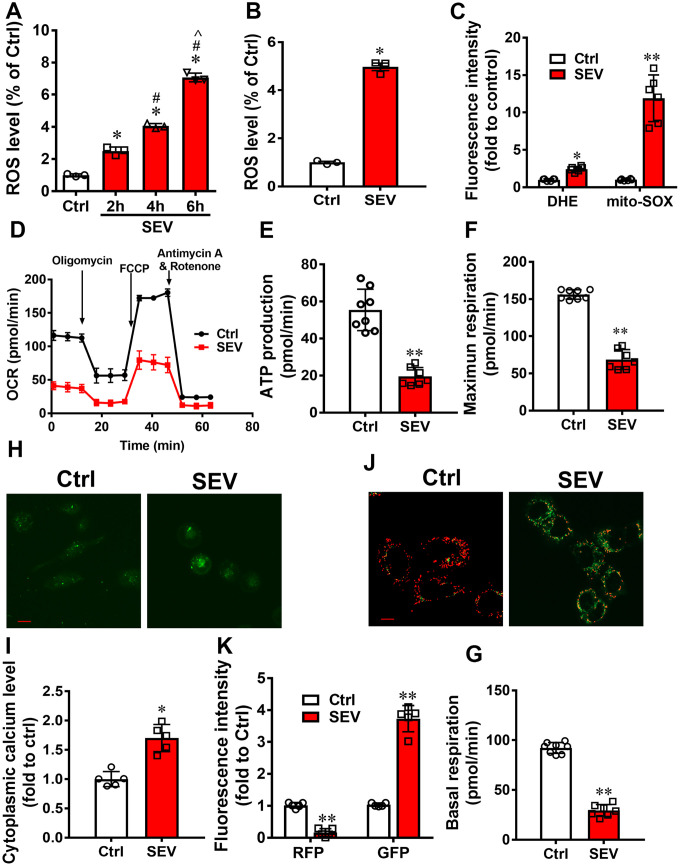
**Sevoflurane induced mitochondria impairment in vitro.** The ROS levels of H4 cells were tested after treatment with 4.1% sevoflurane for 2 h, 4 h, and 6 h (SEV-2h, SEV-4h, and SEV-6h, respectively) (**A**) H4 cells were treated with 4.1% sevoflurane for 6 h and ROS level was measured after 24 h. (**B**) The intracellular ROS and mitochondrial ROS levels were measured after treatment with 4.1% sevoflurane for 6 h (**C**) The OCR assay was used to observe the mitochondrial respiratory function in H4 cells after treatment with 4.1% sevoflurane for 6 h (SEV) (**D**–**G**). The cytoplasmic calcium levels were detected using a Fluo-4 AM probe (**H**, **I**) and the membrane potential was measured using a JC-1 probe (**J**, **K**). Images show representative examples from three independent experiments for each group. The data are expressed as mean ± SD. (A) *P<0.05, Ctrl vs SEV-2h, SEV-4h and SEV-6h group; # P<0.05, SEV-2h vs SEV-4h and SEV-6h group; ^ P<0.05, SEV-4h vs SEV-6h group. (B) * P<0.05, Ctrl vs SEV-6h group. (C, E, F, G, I, K) *P<0.05, **P<0.05, Ctrl vs SEV group.

ROS was assessed after treating H4 cells with sevoflurane for 2 h (SEV-2h), 4 h (SEV-4h) or 6 h (SEV-6h). The results showed that the ROS level was significantly elevated in the SEV-2h, SEV-4h, and SEV-6h groups compared with the Ctrl group, with ROS level increasing with the duration of treatment (Figure 1A; *P*<0.05). The ROS level was dramatically higher in the SEV-6h group than in the Ctrl group at 24 h after sevoflurane administration (Figure 1B; *P*<0.05). Furthermore, we used mito-SOX to detect mitochondrial ROS and DHE to detect intracellular ROS in H4 cells after sevoflurane treatment. The data indicated that sevoflurane increased the intracellular ROS level by 2.4-fold (*P*<0.05) and the mitochondrial ROS level by 11.9-fold (*P*<0.01) (Figure 1C).

Mitochondria are the factories of cellular energy. The energy used to make ATP is actually derived from the mitochondrial membrane potential, a proton chemical gradient that forms across the mitochondrial inner membrane. Because we observed a decline in the mitochondrial membrane potential (Figure 1J, 1K; *P*<0.05) and an increase in cytoplasm calcium levels (Figure 1H, 1I; *P*<0.05) in H4 cells treated with sevoflurane, we then measured the OCR to evaluate mitochondrial respiratory function. Sevoflurane treatment significantly inhibited mitochondrial respiration (Figure 1D–1G; *P*<0.01). All data indicated that sevoflurane disrupted the mitochondria balance in H4 cells.

**Figure 2:**
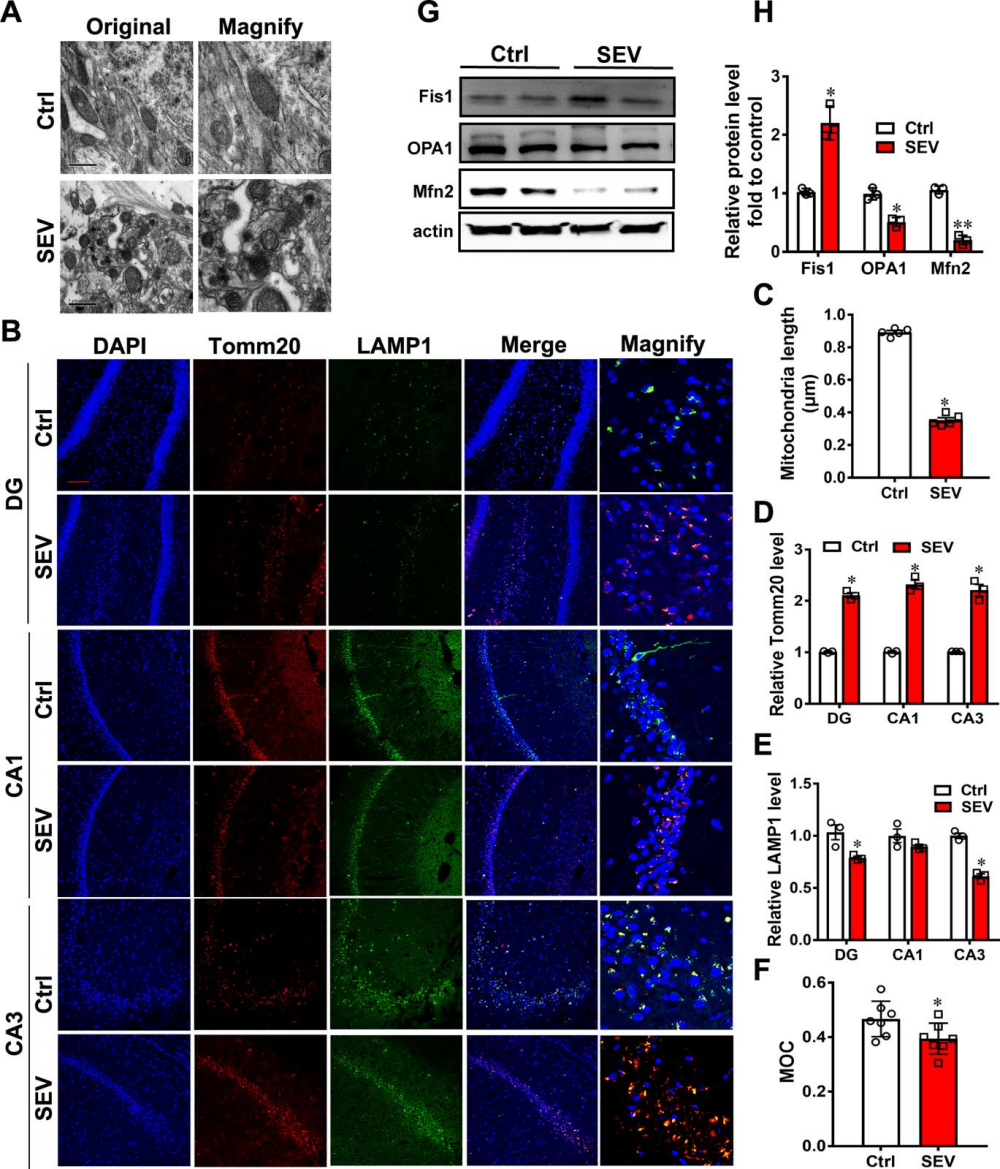
**Sevoflurane induced mitochondria impairment *in vivo***. Eighteen-month-old rats were subjected to 2% sevoflurane for 5 h (SEV). After perfusion, the ultrastructure of mitochondria in the hippocampus was observed under an electron microscope. (**A**) The lengths of all mitochondria were measured and are shown in (**C**) Tomm20 and LAMP1 protein levels in the hippocampus were measured by immunofluorescence assay. Scale bar represents 50 μm. (**B**) The levels of Tomm20 are shown in (**D**) and LAMP1 in (**E**) Manders’ overlap coefficient (MOC) was calculated to determine the degree of colocalization. (**F**) The proteins Fis1, OPA1 and Mfn2 were examined by western blotting. (**G**) The results of semi-quantitative analysis of Fis1, OPA1, Mfn2 and β-actin are shown in (**H**) Images show representative examples from three independent experiments for each group. The data are expressed as mean ± SD. (C-R) * *P*<0.05, **P<0.01, Ctrl vs SEV.

To examine the impact of sevoflurane on aged rats, 18-month-old rats were treated with 2% sevoflurane for 5 h (SEV group), and the mitochondria were observed using cryo-electron microscopy. We observed that the longest diameter of mitochondria was smaller after sevoflurane treatment. (Figure 2A, 2C; *P*<0.05; Supplementary Figure 1C). Moreover, immunofluorescence assays of Tomm20 and LAMP1 abundance revealed that the fluorescence intensity of Tomm20 was significantly elevated, while the fluorescence intensity of LAMP1 was reduced in the DG, CA1 and CA3 regions (Figure 2B). Quantitative analysis showed that the Tomm20 level (in DG, CA1 and CA3) was higher in the SEV group than in the Ctrl group (Figure 2D; P<0.05), while the LAMP1 level (DG and CA3) was lower in the SEV group than in the Ctrl group (Figure 2E; P<0.05). Sevoflurane also decreased the Manders’ overlap coefficient (MOC), which was used to quantify the degree of colocalization between fluorophores [24], for Tomm20 and LAMP1 in the hippocampus of aged rats (Figure 2F). Sevoflurane could not change the mRNA level of Tomm20 and LAMP1 (Supplementary Figure 1B). The expression of Fis1, OPA1 and Mfn2 is are involved in dynamic mitochondrial fusion and fission, which maintain the mitochondrial network in a healthy state. Sevoflurane increased the expression of Fis1 and reduced the expression of OPA1 and Mfn2 in the hippocampus of aged rats (Figure 2G, 2H; P<0.05). Sevoflurane reduced the Mfn2 expression in the hippocampus of aged rats (Supplementary Figure 1B). These data suggested that sevoflurane causes mitochondria impairment in aged rats.

### Sevoflurane induced mitophagy dysfunction

**Figure 3:**
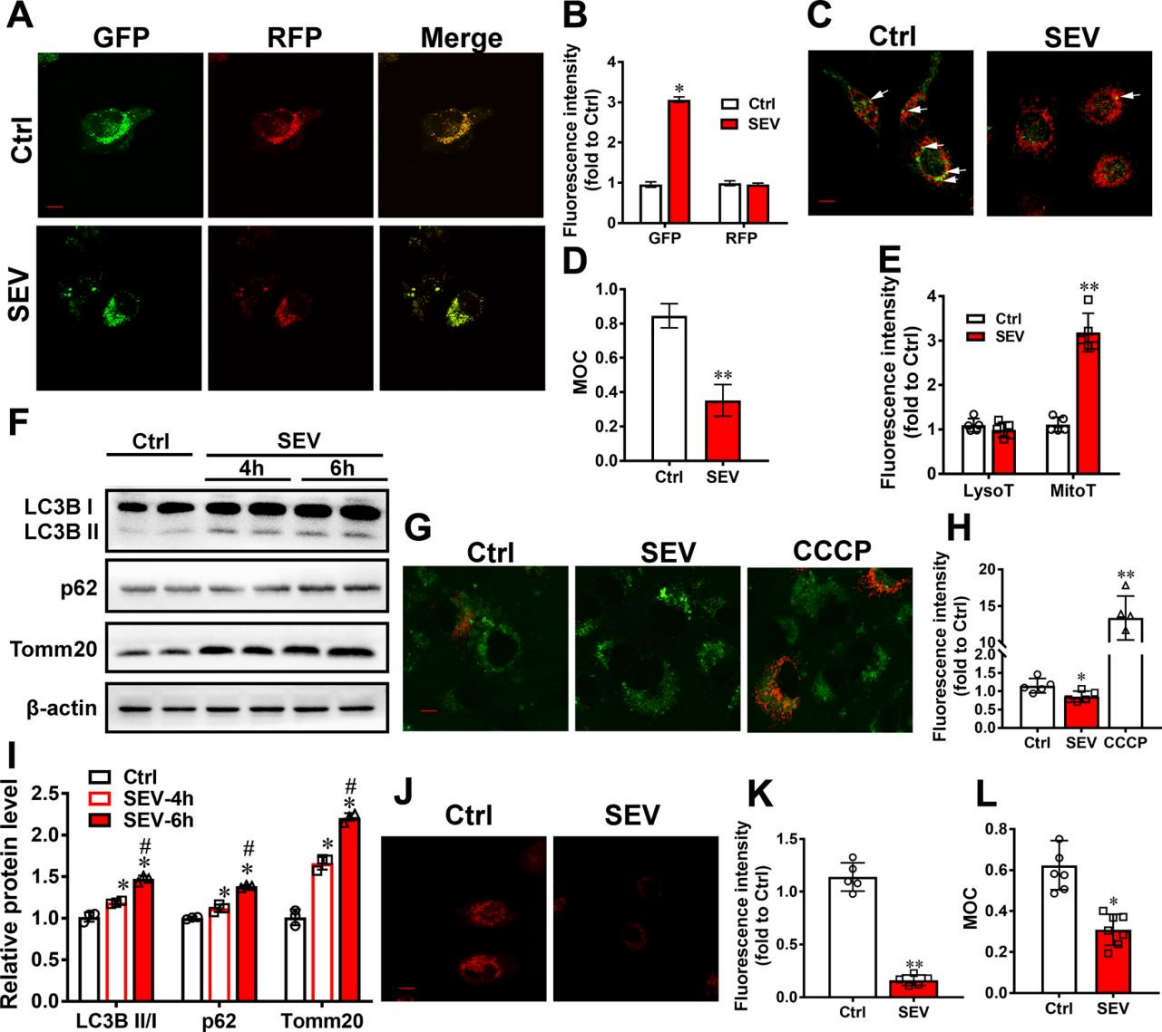
**Sevoflurane induced mitophagy dysfunction *in vitro***. H4 cells were treated for 6 h with 4.1% sevoflurane 24 h after being transfected with AdM-CMV-mCherry-EGFP-LC3B adenoviruses. Fluorescent images were captured by confocal microscopy (**A**). The results of fluorescent analysis (**B**) and Manders’ overlap coefficient (**D**) are shown. H4 cells were treated with 4.1% sevoflurane for 6 h (SEV), and mitochondria and lysosomes were visualized using MitoTracker Red and LysoTracker Green (**C**). The results of fluorescent analysis (**E**) and Manders’ overlap coefficient (**L**) are shown. The LysoT referred to the fluorescent intensity of LysoTracker Green and the MitoT referred to the fluorescent intensity of MitoTracker Red. Primary cultured neurons were subjected to 4.1% sevoflurane for 4 h (SEV-4h) and 6 h (SEV-6h). The LC3B, p62 and Tomm20 protein levels were determined by western blotting analysis (**F**). The results of semi-quantitative analysis of LC3B, p62, Tomm20 and β-actin are shown in (**I**). H4 cells were treated for 6 h with 4.1% sevoflurane and CCCP (50 μM) for 30 min 24 h after being transfected with mt-Keima reporters. Fluorescent images were captured by confocal microscopy (**G**). The results of fluorescent analysis are shown in (**H**). H4 cells were treated with 4.1% sevoflurane for 6 h (SEV), and then treated with DQ-BSA at a final concentration of 10 μg/mL for 30 min. Intracellular fluorescent signals were analyzed by confocal fluorescence microscopy (**J**). The results of fluorescent analysis of DQ-BSA are shown in (**K**). Images show representative examples from three independent experiments for each group. Scale bar represents 10 μm. The data are expressed as mean ± SD. (**B**, **D**, **E**, **K**, **L**) *P<0.05, **P<0.01 Ctrl vs SEV. (**I**) *P<0.05, Ctrl vs SEV-4h and SEV-6h; # *P*<0.05, SEV-4h vs SEV-6h. (**H**) * *P*<0.05, Ctrl vs SEV; ** *P*<0.01, Ctr vs CCCP.

Mitophagy is an evolutionarily conserved cellular process in which dysfunctional or superfluous mitochondria are removed. To investigate whether mitophagy is induced post sevoflurane administration, AdM-CMV-mCherry-EGFP-LC3B adenoviruses were transfected into H4 cells. GFP fluorescence is quenched in the acidic pH of the lysosomal compartment, thereby limiting the use of EGFP-LC3B for the identification of autophagosomes. However, mCherry continues to fluoresce, and mCherry-LC3 can be used to identify both autophagosomes and autolysosomes. Quantification of immunofluoresence showed that the fluorescence intensity of GFP was stronger in the SEV group than in the Ctrl group, but there was no difference in the fluorescence intensity of RFP between these groups (Figure 3A, 3B; *P*<0.05). In addition, sevoflurane decreased the Manders’ overlap coefficient for GFP and RFP. (Figure 3D). Furthermore, we measured mitophagic flux by employing mt-Keima. Addition of the mitophagy inducer CCCP dramatically increased red fluorescence in H4 cells, suggesting that mitophagy was induced. Cells treated with sevoflurane had lower red fluorescence compared with control cells (Figure 3G, 3H; *P*<0.05). These data suggested that sevoflurane causes mitophagy dysfunction in H4 cells.

Because the results from MitoTracker and Lyso Tracker experiments indicated that sevoflurane increased the number of mitochondria but not lysosomes in H4 cells (Figure 3C, 3E, 3L; *P*<0.01), we examined lysosome function after sevoflurane treatment. The lysosomal degradation pathway of macroautophagy (hereafter referred to as autophagy) plays a crucial role in cellular physiology by regulating the removal of unwanted cargoes such as protein aggregates and damaged organelles [25]. We used the DQ-BSA dye to examine whether lysosomal function is impaired upon sevoflurane treatment. We observed that exposure of H4 cells to sevoflurane resulted in a significant reduction of DQ-BSA fluorescence intensity (Figure 3J, 3K; *P*<0.01). These data suggest that sevoflurane also induced lysosomal function impairment.

Next, the protein levels of LC3B, p62 and Tomm20in primary cultured neurons were detected by western blotting. The levels of all three proteins were higher in the SEV-4h and SEV-6h groups compared with the Ctrl group, and higher in the SEV-6h group than in the SEV-4h group (Figure 3F, 3I, *P*<0.05).

**Figure 4:**
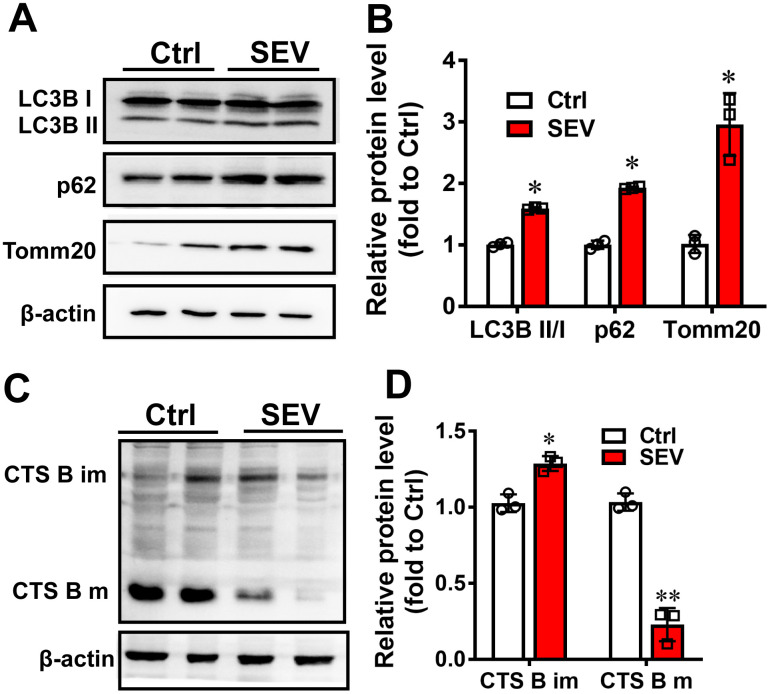
**Sevoflurane induced mitophagy dysfunction *in vivo.*** Eighteen-month-old rats were subjected to 2% sevoflurane for 5 h (SEV). The LC3B, p62 and Tomm20 protein levels in the hippocampus were determined by western blotting analysis (**A**). The results of semi-quantitative analysis of LC3B, p62, Tomm20 and β-actin are shown in (**B**). Cathepsin B (CTS B) and β-actin levels were determined by western blot (**C**). Results of semi-quantitative analysis of Cathepsin B bands are shown in (**D**). The data are expressed as mean ± SD. *P<0.05, **P<0.01 Ctrl vs SEV.

Sevoflurane also impacted mitophagy in vivo. In the hippocampus, LC3B II/I, p62 and Tomm20 expression was also upregulated in the SEV-6h group compared with the Ctrl group (Figure 4A, 4B; *P*<0.05). We also examined the expression of cathepsin B in lysosomes in response to sevoflurane. Cathepsins are the most extensively studied of the lysosomal proteases that participate in autophagic degradation [26]. The results showed that the expression of cathepsin B was lower in the hippocampus of aged rats following sevoflurane treatment. Moreover, the mature forms of cathepsin B almost disappeared after sevoflurane treatment (Figure 4C, 4D; *P*<0.05). These data suggest that sevoflurane also induced lysosomal function impairment. All data suggested that sevoflurane leads to mitochondria accumulation, blocks autolysosome formation and inhibits mitophagic flux.

### Rapamycin relieved sevoflurane-induced mitochondria impairment

**Figure 5:**
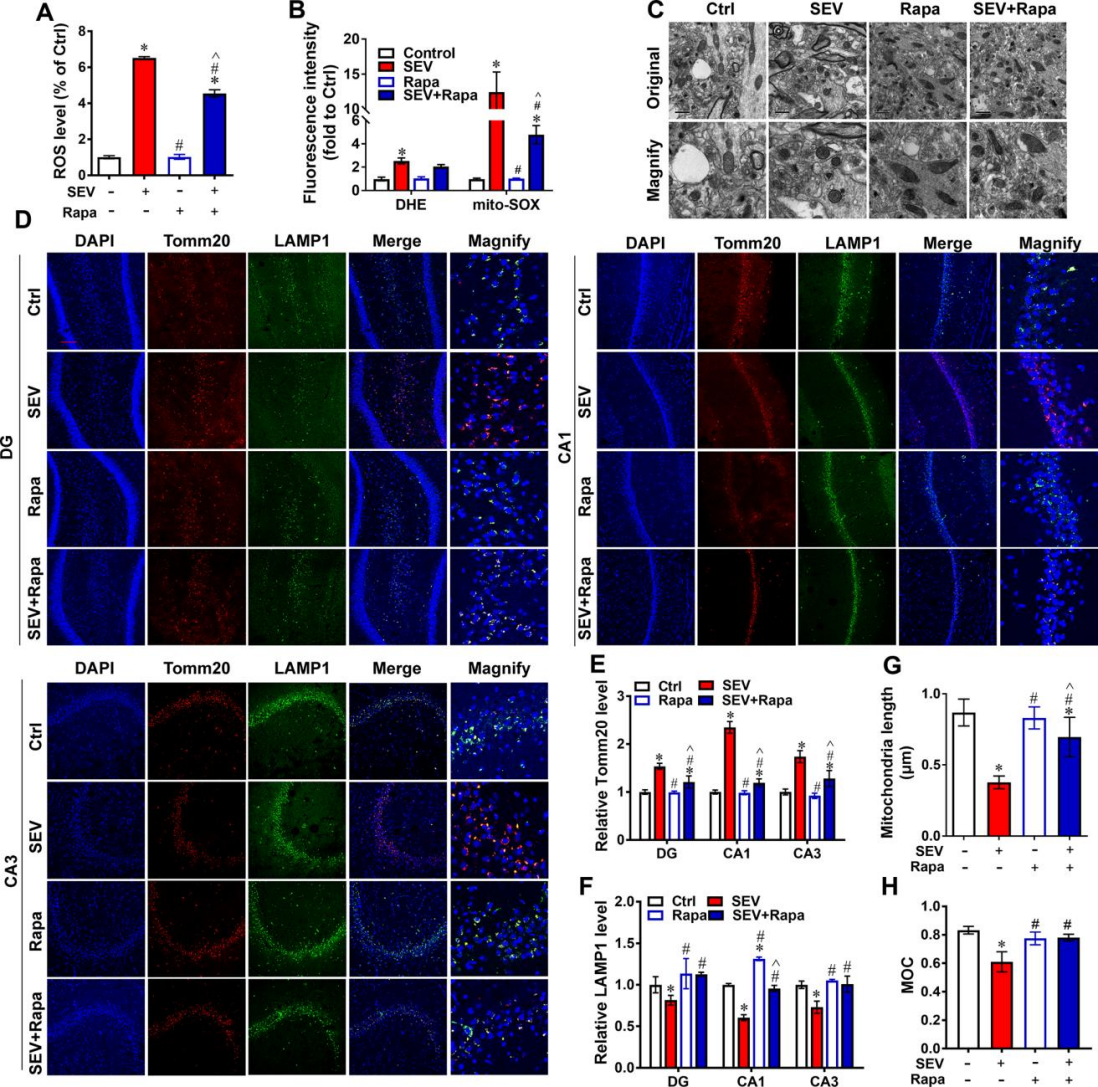
**Rapamycin relieved sevoflurane-induced mitochondria impairment.** H4 cells were exposed to 0% sevoflurane with rapamycin (1μmol/L) (Rapa), 4.1% sevoflurane without rapamycin (SEV), 4.1% sevoflurane with rapamycin (SEV+Rapa), or 0% sevoflurane without rapamycin (Ctrl) for 6 h, and the ROS level (**A**) and intracellular ROS and mitochondrial ROS levels (**B**) were measured. Eighteen-month-old rats were subjected to 2% sevoflurane (SEV and SEV+Rapa groups) for 5 h. Rapamycin (20 mg/kg/d) was administrated intraperitoneally two days before sevoflurane treatment, and daily administrations were continued for one week (Rapa and SEV+Rapa groups). After perfusion, the ultrastructure of mitochondria in the hippocampus was observed under an electron microscope. (**C**) The lengths of all mitochondria were measured. (**G**) The Tomm20 and LAMP1 protein levels were measured by immunofluorescence assay. Scale bar represents 50 μm. (**D**) The results of Tomm20 (**E**) and LAMP1 (**F**) quantification are shown. The Manders’ overlap coefficient is shown in (**H**). Images show representative examples from three independent experiments for each group. The data are expressed as mean ± SD. *P<0.05, Ctrl vs SEV, Rapa and SEV+Rapa; #*P*<0.05, SEV vs Rapa and SEV+Rapa; ^*P*<0.05, Rapa vs SEV+Rapa.

To further confirm the effect of sevoflurane administration on mitophagy, rapamycin, an autophagy inducer, was added to cultured cells. The results showed that the ROS level was significantly higher in H4 cells of the SEV group compared with those of the Ctrl group, and the ROS level in H4 cells was lower in the SEV+Rapa group than in the SEV group (Figure 5A; *P*<0.05). Rapamycin decreased the ROS level after sevoflurane treatment in N2A cells (Supplementary Figure 1A). Sevoflurane also increased both the intracellular ROS level and mitochondrial ROS level, and rapamycin reduced the increase in mitochondrial ROS level after sevoflurane treatment in H4 cells (Figure 5B; *P*<0.05).

Next, eighteen-month-old rats were administrated with rapamycin following sevoflurane treatment. Rapamycin improved the mitochondria morphology in the hippocampus of aged rats, as determined by analysis of reconstructions of mitochondria from cryo-electron microscopy images. The longest diameter of mitochondria was smaller in the SEV group than in the Ctrl group, but was larger in the SEV+Rapa group than in the SEV group (Figure 5C, 5G; *P*<0.05). Immuno-staining assays were also performed to measure the expression of LAMP1 and Tomm20 in the DG, CA1 and CA3 regions. Fluorescence imaging and quantitative data indicated that the fluorescence intensity of LAMP1 was lower and the fluorescence intensity of Tomm20 was higher in the SEV group compared with the Ctrl group in all three regions. In addition, the fluorescence intensity of LAMP1 was higher and the fluorescence intensity of Tomm20 was lower in SEV+Rapa group than in the SEV group in all three regions (Figure 5D–5F; *P*<0.05). Sevoflurane decreased the Manders’ overlap coefficient for Tomm20 and LAMP1 in the hippocampus, and the Manders’ overlap coefficient was higher in SEV+Rapa group than in the SEV group (Figure 5H; *P*<0.05). These results indicated that rapamycin improves mitochondria function after sevoflurane treatment.

### Rapamycin reversed sevoflurane-induced mitophagy dysfunction

**Figure 6:**
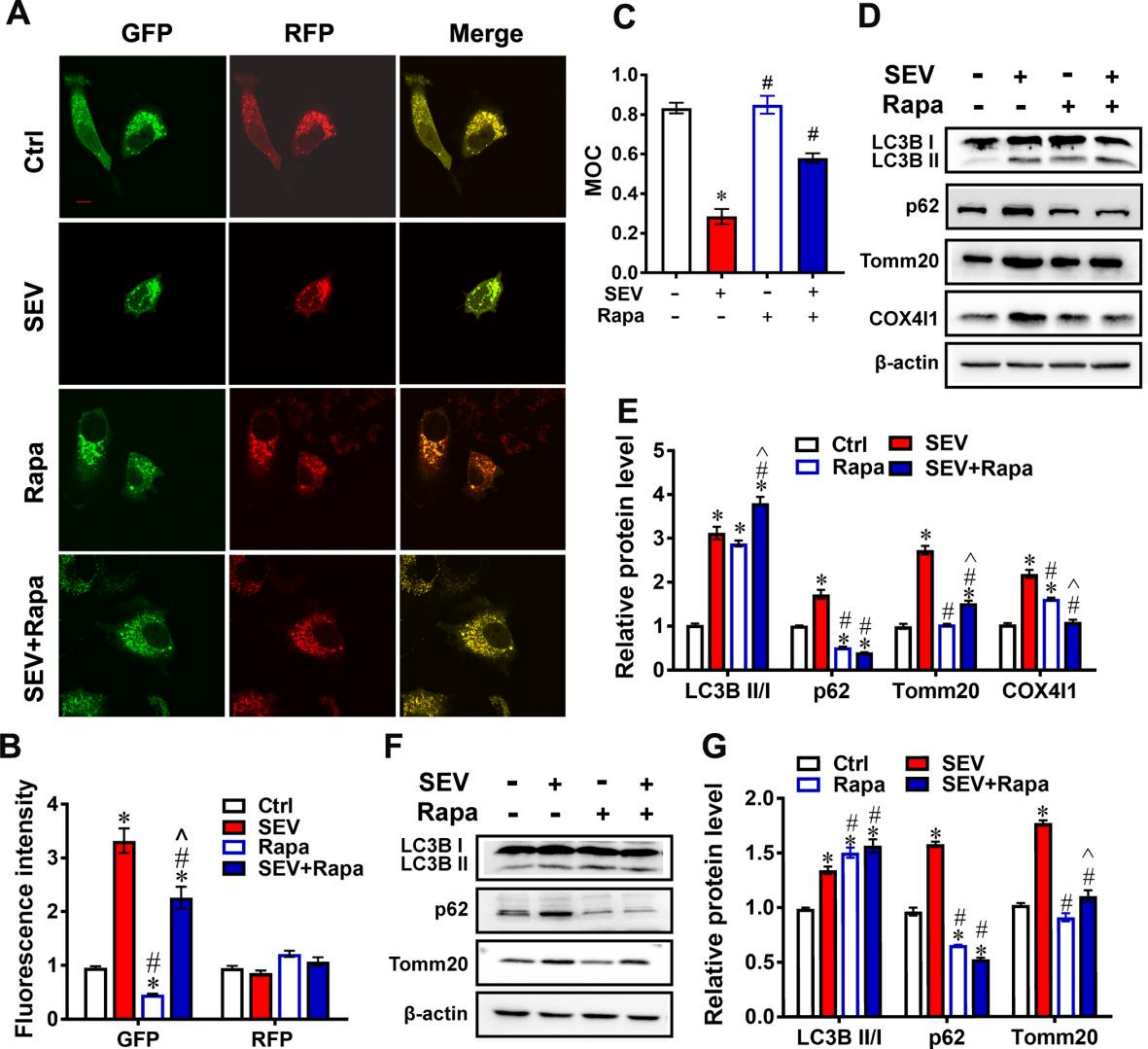
**Rapamycin reversed sevoflurane-induced mitophagy dysfunction.** H4 cells were treated for 6 h with 0% sevoflurane with rapamycin (1μmol/L) (Rapa), 4.1% sevoflurane without rapamycin (SEV), 4.1% sevoflurane with rapamycin (SEV+Rapa), or 0% sevoflurane without rapamycin (Ctrl) 24 h after being transfected with AdM-CMV-mCherry-EGFP-LC3B adenoviruses. Fluorescent images were captured by confocal microscopy (**A**). Images show representative examples from three independent experiments for each group. The results of fluorescent analysis are shown in (**B**). The Manders’ overlap coefficient is shown in (**C**) Primary cultured neurons were subjected to 0% with rapamycin (1 μmol/L) (Rapa), 4.1% sevoflurane without rapamycin (SEV), 4.1% sevoflurane with rapamycin (SEV+Rapa), or 0% sevoflurane without rapamycin (Ctrl) for 6 h. The LC3B, p62, Tomm20 and COX4l1 protein levels were determined by western blotting analysis (**D**). Results of semi-quantitative analysis of LC3B, p62, Tomm20 and COX4l1 are shown in (**E**). Eighteen-month-old rats were subjected to 2% sevoflurane for 5 h (SEV and SEV+Rapa groups). Rapamycin (20 mg/kg/d) was administrated intraperitoneally two days before sevoflurane treatment, and daily administrations were continued for one week (Rapa and SEV+Rapa groups). After perfusion, the LC3B, p62 and Tomm20 protein levels were determined by western blotting analysis (**F**). Results of semi-quantitative analysis of LC3B, p62 and Tomm20 are shown in (**G**). The data are expressed as mean ± SD. *P<0.05, Ctrl vs SEV, Rapa, and SEV+Rapa; # *P*<0.05, SEV vs Rapa and SEV+Rapa; ^ *P*<0.05, Rapa vs SEV+Rapa.

To further clarify whether the inhibition of mitophagy is involved in the sevoflurane-induced mitochondria impairment, AdM-CMV-mCherry-EGFP-LC3B adeno-viruses were used to investigate the expression of autophagy markers. The results showed that the fluorescence intensity of GFP was obviously increased

in the SEV group compared with the Ctrl group, but was lower in the SEV+Rapa group than in the SEV group (Figure 6A, 6B; *P*<0.05). The decrease in GFP fluorescence indicated that rapamycin restored autophagic flux. Next, a western blotting assay was employed to detect the protein levels of the autophagy markers LC3B II/I, p62, Tomm20 and COX4l1 in primary cultured neurons. The results showed that the expression of all four proteins was higher in the SEV group than in the Ctrl group. LC3B II/I expression was higher and p62, Tomm20 and COX4l1 expression was lower in the SEV+Rapa group than in the SEV group (Figure 6D, 6E; *P*<0.05). In the rat hippocampus, LC3B II/I expression was higher in the SEV+Rapa group than in the SEV group, and p62 and Tomm20 expression was lower (Figure 6F, 6G; *P*<0.05). Overall, these results indicated that rapamycin reversed sevoflurane-induced mitophagy dysfunction.

### Rapamycin ameliorated sevoflurane-induced cognitive dysfunction

**Figure 7:**
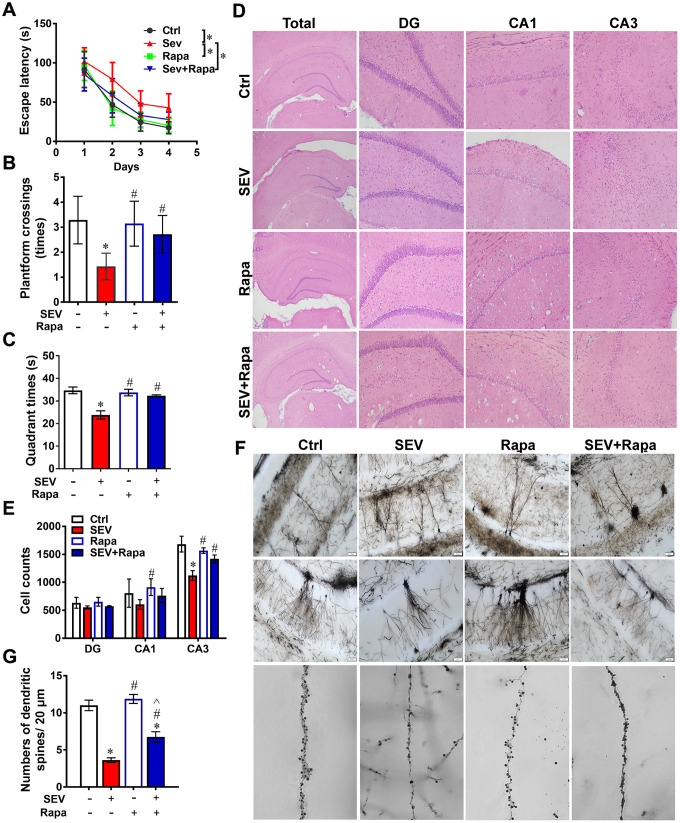
**Rapamycin ameliorated sevoflurane-induced cognitive dysfunction**. Eighteen-month-old rats were subjected to 2% sevoflurane for 5 h (SEV and SEV+Rapa groups). Rapamycin (20 mg/kg/d) was administrated intraperitoneally two days before sevoflurane treatment, and daily administrations were continued for one week (Rapa and SEV+Rapa groups). The Morris Water Maze was used to test memory ability. The parameters escape latency (**A**) number of platform crossings (**B**) and quadrant time (**C**) were measured. Hippocampi were stained with hematoxylin and eosin (**D**) and the number of pyramidal neurons was counted. (**E**) The structure of dendritic spines in the hippocampus are shown in (**F**). The number of dendritic spines in 20 μm at an amplification of 60X were counted for every image. (**G**) Images show representative examples from three independent experiments for each group. The data are expressed as mean ± SD. (**A**) *P*<0.05, Ctrl vs SEV; SEV vs SEV+Rapa. (B, C) (E) (G) *P*<0.05, Ctrl vs SEV, Rapa, and SEV+Rapa; # *P*<0.05, SEV vs Rapa and SEV+Rapa; ^ *P*<0.05, Rapa vs SEV+Rapa.

A behavior test was employed to evaluate the effect of sevoflurane administration on cognitive function of aged rats. The escape latency was significantly prolonged in the SEV group compared with that in the Ctrl group at 1, 2, 3 and 4 days after sevoflurane administration. However, the escape latency was shortened in the SEV+Rapa group compared with that in the SEV group at the same time points (Figure 7A, *P*<0.05). Moreover, the platform crossing times and quadrant times were shorter in the SEV group compared with those in the Ctrl group, but were much longer in the SEV+Rapa group compared with the SEV group (Figure 7B, 7C, *P*<0.05).

Hematoxylin and eosin staining were performed to assess the effect of sevoflurane administration on hippocampal pyramidal neurons. The quantitative data showed that the number of cells in the CA3 region was lower in the SEV group than in the Ctrl group, and higher in the SEV+Rapa group compared with the SEV group. However, there was no significant difference in the number of cells in the DG and CA1 regions (Figure 7D, 7E; P<0.05). The morphology of neuronal dendrites and dendritic spines were observed using Golgi-Cox staining. The dendrite length (measured from the base of the dendrite to the tip of the dendrite) and the number of spines on the dendrite were analyzed. The results showed that the number of dendrites longer than 20 μm was lower in the SEV group compared with the Ctrl group, but higher in the SEV+Rapa group than in the SEV group (Figure 7F, 7G; *P*<0.05). A decline in the number of dendritic spines in the hippocampus and hippocampal pyramidal neurons led to cognitive dysfunction in aged rats. Rapamycin relieved the sevoflurane-induced decrease in the number of hippocampal pyramidal neurons and dendritic spines in the hippocampus and ameliorated cognitive deficits.

### Sevoflurane induced mitophagy dysfunction by inhibiting Parkin expression

**Figure 8:**
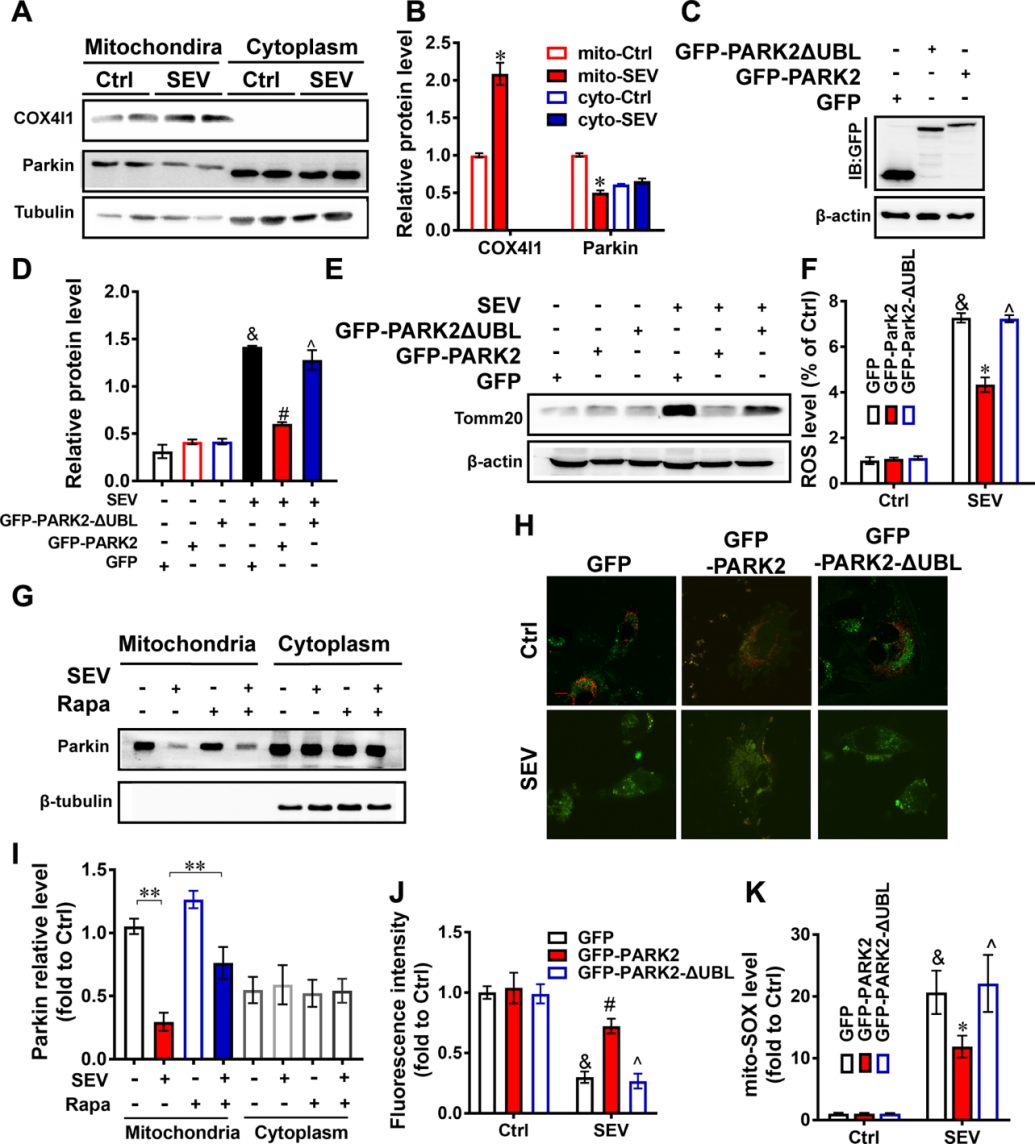
**PARK2 is involved in neuroprotection mediated by mitophagy** Eighteen-month-old rats were subjected to 2% sevoflurane for 5 h. The COX4l1 and Parkin protein levels in both the cytoplasm and mitochondria were detected by western blotting assay (**A**). The mito-Ctrl group and mito-SEV group referred to the mitochondria protein of hippocampus from aged rats with untreated or treated with sevoflurane. The cyto-Ctrl group and cyto-SEV group referred to the cytoplasmic protein of hippocampus from aged rats with untreated or treated with sevoflurane. The results of semi-quantitative analysis of COX4l1, Parkin and Tubulin are shown in (**B**). H4 cells were transfected with plasmids encoding pEGFP, PEGF-PARK2 or pEGF-PARK2ΔUBL following treatment of cells with 4.1% sevoflurane for 6 h. The levels of proteins after transfection were determined by western blotting against GFP (**C**). The Tomm20 level in the indicated groups were examined by western blotting (**E**). The results of semi-quantitative analysis of Tomm20 and β-actin are shown in (**D**). The ROS level (**F**) and mitochondrial ROS level (**K**) of each group were measured. Eighteen-month-old rats were subjected to 2% sevoflurane for 5 h. Rapamycin (20 mg/kg/d) was administrated intraperitoneally two days before sevoflurane treatment, and daily administrations were continued for one week. The levels of Parkin in both the cytoplasm and mitochondria were detected by western blotting assay (**G**). The results of semi-quantitative analysis of Parkin and Tubulin are shown in (**I**). H4 cells were transfected with plasmids encoding pEGFP, pEGFP-PARK2 or pEGFP-PARK2ΔUBL and a mt-Keima reporter following treatment of cells with 4.1% sevoflurane for 6 h. Intracellular fluorescent signals were analyzed by confocal fluorescence microscopy (**H**). The results of fluorescent analysis are shown in (**J**). Images show representative examples from three independent experiments for each group. Scale bar represents 10 μm. The data are expressed as mean ± SD. (**B**) *P<0.05, mito-Ctrl vs mito-Sev, (**D**, **F**, **J**, **K**) &*P*<0.05, GFP-Ctrl vs GFP-SEV; #*P*<0.05 GFP-SEV vs GFP-PARK2-SEV; ^*P*<0.05 GFP-PARK2-SEV vs GFP-PARK2ΔUBL-SEV. (**I**) **P<0.01.

To elucidate the potential mechanism by which sevoflurane administration induces mitophagy dysfunction, western blotting assays were performed to measure Cox4l1 and Parkin protein accumulation in mitochondria and the cytoplasm. H4 cells were treated with 4.1% sevoflurane. The mitochondria proteins and cytoplasmic proteins in the Control group and SEV group were extracted. The results suggested that Cox4l1 protein expression was higher in the mito-SEV group than in the mito-Ctrl group, but that Parkin protein expression was lower in the mito-SEV group (Figure 8A, 8B; *P*<0.05). EGFP-PARK2 plasmid-transfected H4 cells had decreased intracellular and mitochondrial ROS levels after sevoflurane treatment. This protection was not observed in cells transfected with EGFP-PARK2ΔUBL, which encodes a mutant PARK2 protein without ubiquitin E3 ligase activity (Figure 8F, 8K; *P*<0.05). The accumulation of these proteins after transfection was verified by western blotting against GFP (Figure 8C) Consistent with the decrease in ROS levels, PARK2 transfection enhanced mitophagy, as reflected by a decreased Tomm20 level, suggesting that the E3 ligase function of PARK2 is essential for maintaining mitochondria balance in the presence of sevoflurane. (Figure 8D, 8E; *P*<0.05). Rapamycin rescued the expression levels of Parkin in mitochondria in the hippocampus of aged rats upon sevoflurane treatment (Figure 8G, 8I; *P*<0.01). Next, H4 cells were transfected with mt-Keima reporters and plasmids encoding pEGFP, pEGFP-PARK2 or pEGFP-PARK2ΔUBL (loss-of-function mutation) to monitor mitophagic flux after sevoflurane treatment. Overexpression of PARK2 alleviated mitophagy dysfunction induced by sevoflurane (Figure 8H, 8J; *P*<0.05). Therefore, these results indicate that PARK2-induced mitophagy may be required to maintain a healthy between energy and ROS production in mitochondria after sevoflurane treatment.

## DISCUSSION

Although the research on POCD is in full swing, the underlying pathogenesis is still inconclusive. Our previous research revealed that impaired autophagy in hippocampal neurons of aged rats after sevoflurane anesthesia may contribute to cognitive impairment [23]. In this study, we provide the first evidence for mitophagy dysfunction in both *in vitro* and *in vivo* POCD models. Sevoflurane induced a variety of reactions at the cellular level, including a disrupted mitochondrial respiratory network (Figure 1D–1G), increased mitochondria ROS (Figure 1C), perturbed calcium homeostasis (Figure 1H, 1I) and disrupted membrane potential (Figure 1J, 1K), resulting in mitochondria dysfunction in H4 cells. Furthermore, 5 hours after sevoflurane inhalation, Tomm20 accumulation (Figure 2B, 2D) and imbalanced mitochondrial fusion and fission (Figure 2G, 2H) were observed in aged rat hippocampus [27]. Conversely, rapamycin, an autophagy inducer, enhanced mitophagy, improved mitochondrial health and ameliorated the cognitive deficits in POCD rats Overall, our results suggest that sevoflurane-induced mitophagy dysfunction plays a key role in sevoflurane-induced cognitive impairment.

Mitophagy is the primary clearance mechanism for dysfunctional mitochondria, and therefore controls mitochondrial quality [28]. Here we showed that the expression of Tomm20, a mitochondria marker [29], was increased in the SEV group, both *in vitro* and *in vivo* (Figure 2B, 2D, 3F and 4A), suggesting that mitochondria accumulate after sevoflurane treatment. Accordingly, the expression of LAMP1, a lysosome marker, decreased while Tomm20 expression increased in the hippocampus of aged rats after sevoflurane administration, suggesting that the degradation of impaired mitochondria was compromised (Figure 2B, 2D). The data of mt-Kemia indicated sevoflurane causes mitophagy dysfunction in H4 cells (Figure 3G, 3H). These results, combined with observations of MitoTracker and LysoTracker in H4 cells (Figure 3C, 3E), suggest that the accumulation of damaged mitochondria and disruption of mitochondria degradation induced by sevoflurane might be the result of mitophagy dysfunction.

The results of previous studies indicated that sevoflurane upregulates PINK1 and Parkin protein expression in adult mice [30–32]. The underlying mechanism by which sevoflurane regulates the PINK-Parkin pathway is still not clear. Here we found that sevoflurane reduced the level of Parkin in hippocampal mitochondria of aged rats after sevoflurane treatment (Figure 8A). Furthermore, overexpression of wild-type PARK2, but not UBL-deleted mutant PARK2, partly rescued the elevated mitochondrial ROS levels and prevented Tomm20 accumulation and thus promoted mitophagy (Figure 8K, 8E, 8F). Overexpression of PARK2 also rescued mitopahgic flux after sevoflurane treatment in H4 cells (Figure 8H, 8J). We therefore conclude that mitophagy dysfunction may be the potential mechanism underlying sevoflurane-induced cognitive deficits in aged rats.

Autophagy is a vital catabolic process for recycling cellular components under stress conditions [33], and autophagy is involved in sevoflurane-induced cognitive deficit [23]. Our results showed that sevoflurane upregulated the expression of LC3B II/I and p62 in the hippocampus of aged rats (Figure 4A, 4B), which was also reported by other studies [23, 34]. SQSTM1/p62, a protein associated with autophagosomes and degraded in the autolysosome, is commonly regarded as an autophagy substrate protein [35]. The massive increase in LC3BII/I and p62 protein levels indicates that autophagic flux is impaired after sevoflurane treatment. In addition, we found that sevoflurane significantly increased the number of GFP-LC3 dots, which are induced autophagosomes, after mCherry-EGFP-LC3B adenovirus transfection and reduced red fluorescence intensity after mt-Keima reporter transfection in H4 cells, suggesting mitophagic flux impairment (Figure 3A, 3G, 3H). The mitophagy deficiency induced by sevoflurane may therefore be attributed to autophagy impairment in neuronal cells. Furthermore, we observed that exposure of H4 cells to sevoflurane resulted in a significant reduction of DQ-BSA fluorescence intensity, which is an indicator of lysosomal function (Figure 3J, 3K). The expression of cathepsin B was also lower in the hippocampus of aged rats following sevoflurane treatment, suggestive of lysosomal dysfunction after sevoflurane treatment (Figure 4C, 4D). Our data indicate that mitophagy dysfunction and not general autophagy dysfunction is the major cause of sevoflurane-induced cognitive deficit in aged rats.

Rapamycin is applied as an immunosuppressant and has anti-proliferative properties [36]. Rapamycin-based therapy has shown benefits for patients with renal cancer carcinoma, tuberous sclerosis complex, and lymphangioleiomyomatosis-related tumors [37, 38]. Rapamycin was reported to ameliorate POCD by enhancing autophagy [23, 39, 40]; however, it remains undetermined whether this benefit is associated with mitophagy improvement. Here we also found that rapamycin counteracted the increase in Tomm20 expression and decrease in LAMP1 expression in the hippocampus after sevoflurane treatment (Figure 5D–5F). Furthermore, the expression of p62, Tomm20 and COX4l1 was lower in the SEV+Rapa group than in the SEV group both *in vitro* and *in vivo* (Figure 6D–6G). These data imply that rapamycin deceases the number of damaged mitochondria by promoting mitophagy in the hippocampus. We next asked whether rapamycin may reverse sevoflurane-induced neuronal injury. In addition to decreasing the level of p62 both *in vitro* and *in vivo* (Figure 6D–6G) and decreasing the level of GFP-LC3B in adenovirus-transfected H4 cells (Figure 6A, 6B), rapamycin promoted mitophagy, contributing to increased mitochondria quality in neuronal cells, which is essential for autophagy reversion in the hippocampus after sevoflurane treatment [41, 42]. Furthermore, we showed that rapamycin also relieved the sevoflurane-induced mitochondria impairment by measuring mitochondria ROS levels (Figure 5B) and observing mitochondria in TEM micrographs (Figure 5C, 5G). Rapamycin sustained the morphology of mitochondria after sevoflurane treatment by promoting mitophagy in neuronal cells. When eighteen-month-old rats were given rapamycin before sevoflurane treatment, cognitive deficits were ameliorated. In addition, rapamycin significantly ameliorated sevoflurane-induced cognitive deficit, which was accompanied by an increase in the number of hippocampal pyramidal neurons and dendritic spines. According to Golgi staining analysis, most dendritic spines in these four groups were mushroom spines [43, 44]. The presence of mitochondria in more mature spines suggests functional roles for mitophagy in synaptic transmission or synaptic plasticity. A previous study reported that rapamycin exert positive effort on dendritic spine morphology [45]. Consistent with this finding, we found that rapamycin treatment reversed the loss of mature dendritic spines by promoting mitophagy in neuronal cells, which contributed to the amelioration of sevoflurane-induced cognitive deficit (Figure 7F, 7G). Therefore, our data suggest that rapamycin ameliorates the sevoflurane-induced cognitive deficit by increasing the number hippocampal pyramidal neurons and dendritic spines in the hippocampus and maintaining mitochondria health mainly by activating mitophagy in the hippocampus of aged rats.

In the present study, we identified mitophagy dysfunction as a cause of sevoflurane-induced cognitive impairment. This mitophagy deficiency may be the result of autophagy impairment in neuronal cells. We found that rapamycin ameliorated cognitive deficit in association with its function in reversing mitophagic flux. Although the underlying mechanism of the regulation of mitophagy by sevoflurane needs to be further elucidated, the data on the beneficial effects of rapamycin on memory may shed light on novel interventional strategies for POCD caused by sevoflurane. Restoration of neuronal mitophagy might be a new strategy to rescue POCD-related cognitive deficit.

## MATERIALS AND METHODS

### Animals

Eighteen-month-old rats (550 g-750 g) were used in this study and obtained from Zhejiang Academy of Medical Sciences. The rats were housed in the standard animal care facility for one month under conventional conditions: 12 h/12 h light/dark cycle and 22 °C. Rats had free access to food and water. All experiments were approved by and conducted in accordance with the ethical guidelines of the Zhejiang University Animal Experimentation Committee and were in complete compliance with the National Institutes of Health Guide for the Care and Use of Laboratory Animals. Efforts were made to minimize any pain or discomfort, and the minimum number of animals was used.

### Animal treatment

The rats were randomly assigned to four groups: control group (Ctrl), sevoflurane group (SEV), rapamycin group (Rapa) and sevoflurane plus rapamycin group (SEV+Rapa). To induce general anesthesia, rats were placed in an acrylic anesthetizing chamber with two interfaces: one was connected to a sevoflurane vaporizer and the other was connected to a multi-gas monitor. Rats in the SEV and Sev+Rapa groups were exposed to 2% sevoflurane delivered in humidified 30% O_2_ carrier gas for 5 h. Rats in the Ctrl and Rapa groups were exposed to humidified 30% O2 balanced by N2 in an acrylic anesthetizing chamber without sevoflurane for the same period. Rapamycin was dissolved in dimethylsulfoxide (25 mg/mL) and further diluted in a solution containing 5% polyethylene glycol 400 and 5% Tween 80. Rats in the Rapa and SEV+Rapa groups were injected daily with rapamycin (20 mg/kg/d) intraperitoneally, and rats in the Ctrl and SEV groups were injected with a vehicle solution (5% polyethylene glycol 400 and 5% Tween 80). Daily injections started two days prior to the start of the anesthesia treatment for all groups, and the injections continued for one week after the first injection for all groups. To ensure sufficient ventilation, a single sample (100 μL) of arterial blood was obtained at the end of sevoflurane anesthesia or sham exposure via cardiac puncture from five rats of each group. These rats were not used for any other part of the study. Arterial carbon dioxide partial pressure (PaCO_2_), arterial oxygen pressure (PaO_2_), blood oxygen saturation (SaO_2_) and power of hydrogen (pH) were measured using a blood gas analyzer (Kent Scientific Corp., Torrington, CT, USA) (Supplementary Table 1). There was no significant difference in pH, PaCO_2_, PaO2, Glucose or SaO_2_ level among the four groups.

### Morris water maze test

After the sevoflurane exposure and rapamycin administration, the spatial memory abilities of rates were tested using the Morris Water Maze (MWM). A circular black pool (diameter, 180 cm; depth, 50 cm) was filled with warm water (25 °C) to a depth of 30 cm, which was made opaque by the addition of black non-toxic ink. An invisible platform (diameter, 10 cm) was fixed in one of the four quadrants of the pool and was submerged 1 cm below the water line. All rats received four training trials every day in each quadrant of the pool. In each trial, the rats were placed into the pool at a random starting position in the quadrant facing the wall of the pool. They were allowed to swim and discover the hidden platform for 120 s. Rats who failed to locate the platform within 2 min were gently guided to the platform. When the rats arrived at the platform, they were allowed to stay on it for 30 s. The latency time (the time to find the hidden platform) was recorded. The average time across the four trials was regarded as the result for a given rat for that day. After each trial, the rat was wiped dry before being returned to its cage. The interval between two trials was greater than 30 min. On the fifth day, the platform was removed and each rat was allowed to swim freely in the pool for 2 min. The number of times that the rat crossed the original platform site and the time spent in the target quadrant were recorded.

### Cell culture

Primary hippocampal neurons were cultured as previously described [46]. Briefly, the dissected hippocampi from E17 fetal mice were treated with 0.125% trypsin in Hank’s buffer (in mmol/L: 137 NaCl, 5.4 KCl, 0.4 KH_2_PO_4_, 0.34 Na_2_PO_4_·7H_2_O, 10 glucose and 10 HEPES) for 12 min at 37 °C and dissociated by repeated passage through a series of fire-polished Pasteur pipettes. Approximately 2 × 10^5^ cells/cm^2^ were seeded onto poly-l-lysine (10 μg/mL)-coated plates. The neurons were grown in Neurobasal Medium (Invitrogen) supplemented with 2% B27 (Invitrogen), 10 U/mL penicillin, 10 U/mL streptomycin, and 0.5 mmol/L glutamine at 37 °C in a humidified atmosphere with 5% CO_2_. Cultures were maintained for 20 d before treatment and harvested for subsequent experiments.

H4 human neuroglioma cells purchased from the China Center for Type Culture Collection were cultured in Dulbecco’s Modified Eagle’s Medium (DMEM) containing 10% heat-inactivated fetal bovine serum and 10% F12 (all from Gibco, Grand Island, NY, USA) at 37 °C with 5% CO_2_ in a humidified incubator.

### Cell exposure to sevoflurane

Culture plates were placed into an airtight plastic chamber (MIC-101) with inlet and outlet connectors. The inlet port of the chamber was connected to a sevoflurane vaporizer to adjust the concentration of sevoflurane. The chamber was then gassed with 4.1% sevoflurane in the carrier gas (95% air/5% CO_2_) for 15 min. The concentration of sevoflurane in the chamber was monitored at the chamber outlet port by a gas monitor (PM 8060, Drager, Lübeck, Germany) until the target concentration was reached. The chamber was then sealed and incubated at 37 °C for 6 h. The gas in the chamber was renewed every 3 h, and the target concentration of sevoflurane was confirmed at the end of the incubation by the gas monitor. The control cells underwent the same procedure but with air containing 5% CO_2_.

### Reactive oxygen species determination

H4 human neuroglioma cells were cultured in 48-well plates. A dosage of 5 μmol/L 2,7-Dichlorodihydro-fluorescein diacetate (DCFH-DA; Sigma, D6883) was added to the cell cultures immediately after treatment. The intracellular fluorescence was detected by a microplate reader with an excitation wavelength of 488 nm and emission wavelength of 520 nm (DTX880, Beckman Coulter, USA). These experiments were repeated three times independently.

### Immunoblotting

Rat brain tissues and H4 cells were homogenized in RIPA buffer (Beyotime, P0013B) with 1× protease inhibitor cocktail (Beyotime, P1010). For mitochondrial protein extraction, a cytoplasmic and mitochondrial protein extraction kit (Sangon Biotech, C500051) was used. The supernatant was collected by centrifugation at 16,200×g for 10 min, and the protein concentration was determined using a bicinchoninic acid protein assay kit (Beyotime, P0012S). An aliquot of 50 μg protein from each sample was separated using SDS-PAGE and transferred to a nitrocellulose membrane, which was then blocked with 5% nonfat milk in phosphate-buffered saline (PBS, pH 7.4). The membranes were incubated with primary antibodies against LC3B (1:1,000; Sigma, L7543), p62 (1:1,000; MBL, PM045), COX4I1 (1:1,000; ABclonal, A6564), Tomm20 (1:1,000; ABclonal, A6774), Parkin (1:1,000; Santa Cruz Biotechnology, sc-32282), Fis1 (1:500; ABclonal, A5821), OPA1 (1:500; ABclonal, A9833), Mfn2 (1:500; ABclonal, A12771), CTSb (1:1,000; abcam, ab58802), actin (1:5,000; ABclonal, AC026) or tubulin (1:10000; Sigma, T9026) at 4 °C overnight. Blots were incubated in horseradish peroxidase-conjugated secondary antibodies against rabbit or mouse IgG (1:5,000, CST, 7071 and 7072) for 2 h at room temperature, then subjected to chemiluminescent detection using the SuperSignal West Pico Substrate (34077, Pierce) and exposed to film. Digital images were quantified using densitometric measurements obtained using Quantity One software (Bio-Rad).

### Transmission electron microscope observation

The tissues from the hippocampus were fixed with 2.5% glutaraldehyde overnight at 4°C. After three rinses with PBS, the tissues were post-fixed with 1% osmium tetraoxide for 2 h. The tissues were then rinsed with distilled water, followed by a graded ethanol dehydration series ending with propylene oxide. After infiltration in a mixture of one-half propylene oxide and one-half resin, the tissues were embedded in resin. Sections (120 nm) were cut and stained with 4% uranylacetate for 20 min and with 0.5% lead citrate for 5 min. Mitochondria in the hippocampal neurons were observed on a transmission electron microscope (TEM) (Phliphs Tecnai 10, Holland) in the Center of Cryo-Electron Microscopy at Zhejiang University.

### Immunostaining

Rats were anaesthetized with isoflurane and perfused with 0.1 mol/L PBS (pH 7.2-7.4) via the ascending aorta followed by perfusion with 4% paraformaldehyde in 0.1 mol/L Phosphate Buffer (PB). The brains were then removed, and post-fixed in the same fixative for 2 h before cryoprotection in PB containing 15% sucrose at 4°C for 2 days. The brains were then transferred into PB containing 30% sucrose at 4°C for another 2 days for dehydration. The brains were wrapped and embedded in aluminum foil with O.C.T compound and stored at -70°C until sectioning was performed. Serial cryostat sections, 25 μm thick, of the hippocampus region were collected.

Sections were incubated with 5% donkey serum, 0.3% Triton X-100 for 1 h at room temperature, then with antibody diluent containing goat antibodies against Tomm20 (1:50; ABclonal, A6774) and LAMP1 (1:200; ABclonal, A16894) for 1 day at 4°C. Then sections were rinsed with PBS (3×10 min) followed by incubation with Alexa Fluor™ 488 goat anti-mouse antibody and Alexa Fluor™ 594 goat anti-rabbit antibody for 1 h at room temperature. After rinsing with PBS (6×5 min), fluorescence signals were visualized under an epifluorescence microscope. Images were captured with the assistance of Image-Pro Plus 5.0 software, and all the parameters used were kept consistent during capturing.

### Transfection with adenoviruses

AdM-CMV-mCherry-EGFP-LC3B adenoviruses were purchased from Vigene (Shangdong, China). After culturing H4 cells for 24 h, adenovirus-directed gene transfer was performed by adding a small volume of fetal bovine serum-free DMEM containing the constructed adenovirus at a multiplicity of infection of 50 for 6 h. All experiments were performed after 36 h of adenoviral infection. The cells were visualized under an epifluorescence microscope immediately after treating the cells. Images were captured with the assistance of Image-Pro Plus 5.0 software, and all the parameters used were kept consistent during capturing.

### Golgi-Cox staining

The morphology of neuronal dendrites and dendritic spines in the brains of mice were observed by Golgi-Cox staining, which was performed using the Hito Golgi-Cox OptimStain^TM^ PreKit (Hitobiotec Corp. Kingsport, TN, USA). Briefly, following sacrifice of the mice, brain tissue was carefully removed as quickly as possible. The intact brain tissues were rinsed with Milli Q water and impregnated with equal volumes of Solutions A and B from the kit. The impregnation solution was replaced the following day and the tissues stored at room temperature in the solution in darkness for 2 weeks. Brain tissues were then transferred to Solution C. Solution C was replaced the following day and brains further stored at 4 °C for 72 h in the dark. The brains were slowly immersed in cooled isopentane for 1 min, wrapped in aluminum foil and stored at -70°C until sectioning was performed. The brain sections (100 μm thickness) were generated using a cryostat with the chamber temperature set at -19°C.

Each section was mounted on gelatin-coated microscope slides using Solution C. Excess solution was removed from the slide using a Pasteur pipette and further absorbed with stacks of filter paper. The sections were allowed to dry naturally at room temperature (3 days). The dried brain sections were processed as per the manufacturer's instructions. Briefly, dendrites within the CA1 subregion of the hippocampus were imaged using the 20× and 60× objectives of an Olympus BX61. Dendritic spines were detected along CA1 secondary dendrites starting from their point of origin on the primary dendrite, and counting was performed by an experimenter blinded to the group of each sample.

### Hematoxylin and eosin staining

The brain sections were harvested as described above. The brain sections were rinsed with methanol for 1 min and washed with water, followed by incubation with hematoxylin solution for 5 min and eosin solution for 20 sec. The brain sections were mounted in resinene after dehydration and dimethylbenzene treatment. Images were captured with the assistance of Image-Pro Plus 5.0 software using an Olympus BX53, and all the parameters used were kept consistent during capturing.

### Plasmid and transfection

pEGFP-PARK2, expressing full-length mouse PARK2 (RefSeq #: NM_016694.3) was generated by PCR amplification of the *PARK2* sequence from a mouse cDNA library using the primers 5’-GCCAGATCTATGATAGTGTTTGTCAGGTTC-3’ and 5’-GTTGAATTCCTACACGTCAAACCAGTGATC-3’ and cloning the product in-frame into the BglII and EcoRI sites of the Pegfp-C1 vector. pEGFP-PARK2ΔUBL, containing the PARK2 promoter with a deletion in PARK2 corresponding to the sequences from 1 kb to 76 kb in N terminus, was constructed as described for pEGFP-PARK2 except that the primers 5’-GTTGAATTCAGGTCAAGGCTTGGTGGTCTTC-3’ and 5’-AGATGCATTTGTTTCATGACTAGATCTGGC-3’ were used. The plasmids pEGFP, pEGFP-PARK2 and pEGFP-PARK2ΔUBL were transfected into H4 cells using Lipofectamine 3000 reagent following the specific protocol for this cell line.

### Detection of mitochondrial membrane potential

JC-1 (Thermo Fisher Scientific, MA, USA) fluorescent dye was used for monitoring mitochondrial membranes. H4 cells were cultured and treated with or without 4.1% sevoflurane for 6 h, then stained with 10 μM JC-1 for 20 min. JC-1 exhibits fluorescence, either as red fluorescent J-aggregates (530 nm excitation/590 nm emission) at high potentials or as green fluorescent J-monomers (490 nm excitation/530 nm emission) at low potentials. The cells were visualized under an epifluorescence microscope immediately after treatment. Images were captured with the assistance of Image-Pro Plus 5.0 software, and all the parameters used were kept consistent during capturing.

We measured cytoplasmic calcium level using Fluo calcium indicators (Fluo-4, AM, F14201, Invitrogen). An aliquot of DMSO stock solution (5 mM) was diluted to a final concentration of 5 μM in buffered physiological medium. H4 cells were treated with 4.1% sevoflurane for 6 h, then washed in indicator-free medium. Cells were incubated with the fluo acetoxymethyl ester for 30–60 min at 37°C. Before fluorescence was measured, cells were washed in indicator-free medium. The fluorescence signals from the fluo acetoxymethyl ester were visualized under an epifluorescence microscope immediately after treatment. Images were captured with the assistance of Image-Pro Plus 5.0 software, and all the parameters used were kept consistent during capturing.

### Mitochondrial respiration analysis

The oxygen consumption rate (OCR) was assessed using a Seahorse XF96 analyzer (Seahorse Agilent, USA) in combination with the Agilent Seahorse XFe96 Extracellular Flux Assay Kit according to the manufacturer’s recommendations. H4 cells were seeded in 96-wells of a Agilent Seahorse XF96 cell culture microplate (101085-004) and grown to 70% confluence in 200 μL of growth medium prior to analysis. H4 cells were cultured and treated with or without 4.1% sevoflurane for 6 h. On the day of the assay, culture medium was replaced with 175 μL assay mediums, D5030), supplemented with 25 mM glucose, 2 mM glutamine, and 2 mM pyruvate. Prior to the assay, plates were incubated at 37°C for 1 h without CO_2_. Thereafter, basal OCR was measured followed by the automated injection of 25 μl oligomycin (8 μM), mixing for 3 min, and OCR measurement for 2 min. Next, 25 μl carbonyl cyanide 4-(trifluoromethoxy) phenylhydrazone (FCCP) (9 μM) was injected, and after 3 min of mixing OCR was measured for 2 min. Finally, a combination of 25 μl rotenone (20 μM) + antimycin A (100 μM) was injected, followed by the same mixing and measurement steps. After the assays, plates were saved for protein assays, and OCR was normalized to the total protein amount per well.

### Detection of mitophagy using mt-Keima

Keima is a pH-sensitive, dual-excitation fluorescent protein that also exhibits resistance to lysosomal proteases [47]. At physiological pH in the mitochondria (pH 8.0), the shorter-wavelength excitation form predominates. Within acidic lysosome (pH 4.5) after mitophagy, mt-Keima undergoes a gradual shift to the longer-wavelength excitation form. After culturing H4 cells for 24 h, mt-Keima reporters were transfected into H4 cells by adding Lipofectamine 3000 (Thermo Fisher, USA, L300015) and incubating for 8 h. All experiments were performed 16 h after liposome infection. H4 cells were treated with or without 4.1% sevoflurane for 6 h and CCCP (50 μM) for 30 min to induce mitophagy. The cells were visualized under an epifluorescence microscope immediately after treatment. Images were captured with the assistance of Image-Pro Plus 5.0 software, and all the parameters used were kept consistent during capturing. The red fluorescence signal from the untreated cells treated with CCCP dramatically increased, suggesting that mitophagy was induced.

### Measurement of mitochondrial reactive oxygen species

We measured regional mitochondrial ROS accumulation using the Mito-SOX reagent (M36008, Thermo Fisher, USA). After treatment, H4 cells were washed with HBSS solution buffer. A 5 μM Mito-SOX working solution was then prepared. Next, 1.0 mL of the 5 μM Mito-SOX reagent was applied as a cell loading solution in which cells were incubated for 10 min at 37 °C without light exposure. Cells were then gently washed three times with warm PBS. Excitation wavelengths were measured at 510 nm and emission at 580 nm by a fluorescence microplate reader (SpectraMax M5/M5e). The intracellular ROS levels in H4 cells were measured with the fluorescent probe dihydroethidium (DHE). After being treated with 4.1% sevoflurane for 6 h, H4 cells were incubated with 1 μM DHE (Bestbio, China) for 60 min at 37°C. Excitation wavelengths were measured at 518 nm and emission at 610 nm by a fluorescence microplate reader (SpectraMax M5/M5e).

### MitoTracker and LysoTracker imaging

H4 cells were treated with 4.1% sevoflurane for 6 h, then the medium was removed from the dish and replaced with prewarmed (37°C) 50 nM MitoTracker medium for 10 min or 100 nM LysoTracker medium for 1 h. Then the loading solution was replaced with fresh medium. The cells were visualized under an epifluorescence microscope immediately after treatment. Images were captured with the assistance of Image-Pro Plus 5.0 software, and all the parameters used were kept consistent during capturing.

### DQ Red-BSA trafficking assay

Lysosomal proteases were assayed for their ability to process DQ-BSA Red (Invitrogen, D-12051), a protein heavily labeled with a fluorescent BODIPY dye that is self-quenched. Once DQ-BSA enters lysosomal compartments, it is cleaved by lysosomal proteases, resulting in the unquenching and release of bright fluorescent fragments. Thus, the fluorescence intensity of DQ-BSA can be used to visualize intracellular proteolytic activity [48, 49]. After differentiation, H4 cells were incubated with DQ-Red BSA at a concentration of 10 μg/ml in culture medium for 2 h at 37 °C and 5% CO_2_, then the medium was replaced with fresh medium and cells were treated with 4.1% sevoflurane as aforementioned.

### Statistical analysis

SPSS 19.0 software was used to process the data. All data are represented as mean ± standard deviation, and were analyzed by one-way analysis of variance (ANOVA) and Tukey's post hoc test. *P*<0.05 was considered statistically significant.

## Supplementary Material

Supplementary Figure 1

Supplementary Table 1
