# Supplementary material for: Mitophagy impairment is involved in sevoflurane-induced cognitive dysfunction in aged rats
Source: Aging (Albany NY). 2020 Sep 9;12(17):17235–56. doi: 10.18632/aging.103673 (PMC7521530; doi:10.18632/aging.103673)
Supplement: Supplementary Figure 1 [file aging-12-103673-s002..pdf]

SUPPLEMENTARY FIGURE

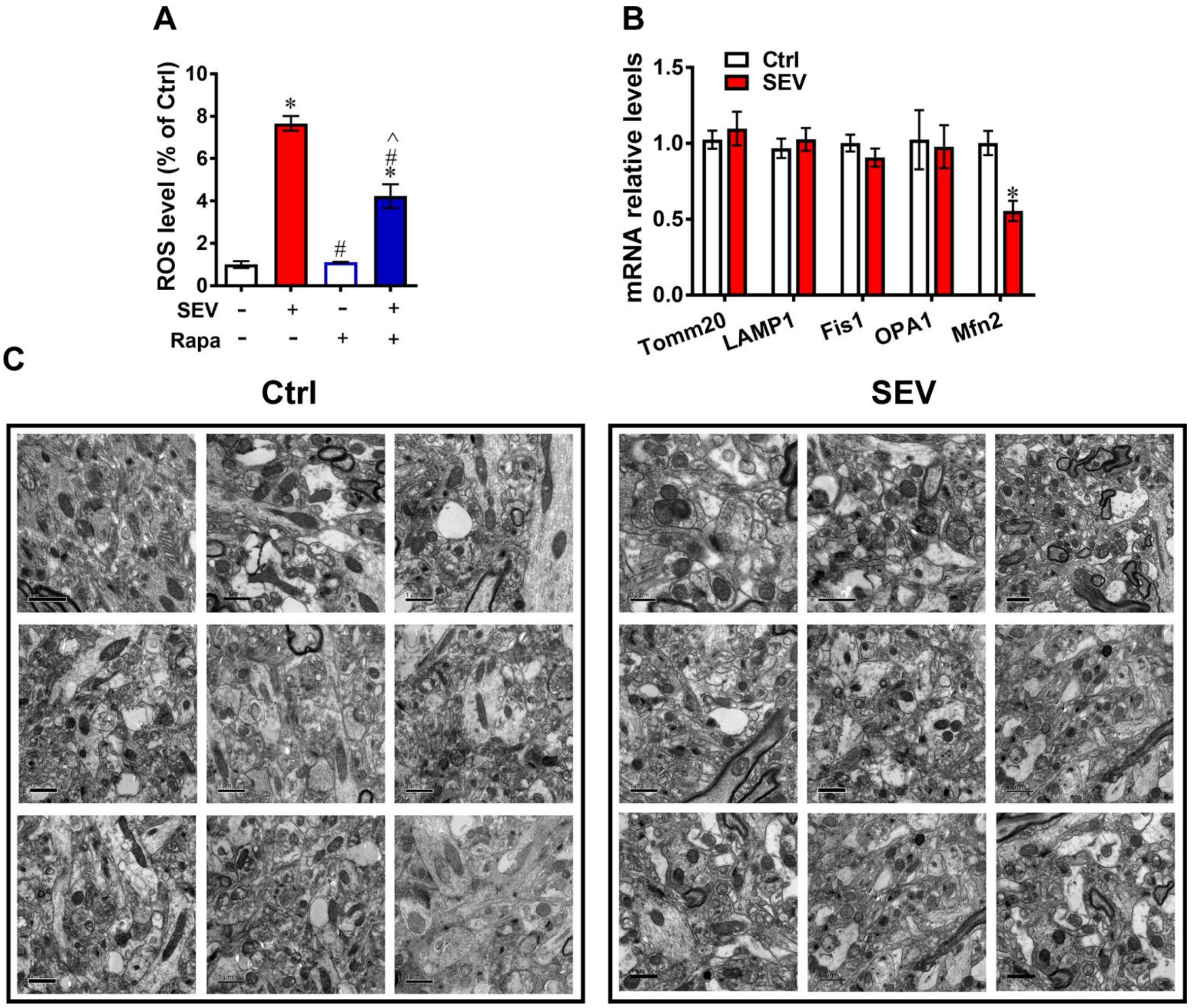

Supplementary Figure 1. N2A cells were exposed to 0% or 4.1% sevoflurane with/without rapamycin (1 μmol/L) for 6 h, and the ROS levels were measured. (**A**) Eighteen-month-old rats were subjected to 2% sevoflurane for 5 h SEV). The mRNA levels of Tomm20, LAMP1, Fis1, OPA1 and Mfn2 in the hippocampus were determined by RT-PCR. (**B**) After perfusion, the ultrastructure of mitochondria in the hippocampus of aged rats was observed under an electron microscope. (**C**) Images show representative examples from three independent experiments for each group. The data are expressed as mean ± SD. **P*<0.05, Ctrl vs SEV; #*P*<0.05, SEV vs Rapa and SEV+Rapa; ^*P*<0.05, Rapa vs SEV+Rapa.
