# Supplementary material for: Mitophagy impairment is involved in sevoflurane-induced cognitive dysfunction in aged rats
Source: Aging (Albany NY). 2020 Sep 9;12(17):17235–56. doi: 10.18632/aging.103673 (PMC7521530; doi:10.18632/aging.103673)
Supplement: Supplementary Table 1 [file aging-12-103673-s001..pdf]

SUPPLEMENTARY TABLE

Supplementary Table 1. Effect of sevoflurane exposure on physiological parameters of arterial blood gas analysis of aged rats.

Group	pH	PaCO2(mmHg)	PaO2(mmHg)	Glucose(mmol/L)	SaO2(%)
Control	7.33±0.03	41.2±3.3	111±13	4.2±0.3	99.0±0.6
Rapa	7.32±0.04	39.3±3.1	109±11	4.0±0.4	99.0±0.5
Sev	7.33±0.01	40.7±3.4	112±14	3.9±0.5	99.0±0.7
Sev+Rapa	7.34±0.05	41.6±3.5	108±13	4.4±0.5	98.0±1.3

PaO2, arterial oxygentension; PaCO2, arterial carbon deoxidate tension; SaO2, arterial oxygen saturation.
